# Aneurysmal coronary artery disease: An overview

**DOI:** 10.21542/gcsp.2017.26

**Published:** 2017-10-31

**Authors:** Mohamed S. ElGuindy, Ahmed M. ElGuindy

**Affiliations:** 1 Department of Cardiology, Cairo University, Egypt; 2 Department of Cardiology, Aswan Heart Centre, Egypt; 3 Imperial College London, UK

## Abstract

Aneurysmal coronary artery disease (ACAD) comprises both coronary artery aneurysms (CAA) and coronary artery ectasia (CAE). The reported prevalence of ACAD varies widely from 0.2 to 10%, with male predominance and a predilection for the right coronary artery (RCA). Atherosclerosis is the commonest cause of ACAD in adults, while Kawasaki disease is the commonest cause in children and adolescents, as well as in the Far East. Most patients are asymptomatic, but when symptoms do exist, they are usually related to myocardial ischemia. Coronary angiography is the mainstay of diagnosis, but follow up is best achieved using noninvasive imaging that does not involve exposure to radiation. The optimal management strategy in patients with ACAD remains controversial. Medical therapy is indicated for the vast majority of patients and includes antiplatelets and/or anticoagulants. Covered stents effectively limit further expansion of the affected coronary segments. Surgical ligation, resection, and coronary artery bypass grafting are appropriate for large lesions and for associated obstructive coronary artery disease.

## Introduction

**Figure 1.**
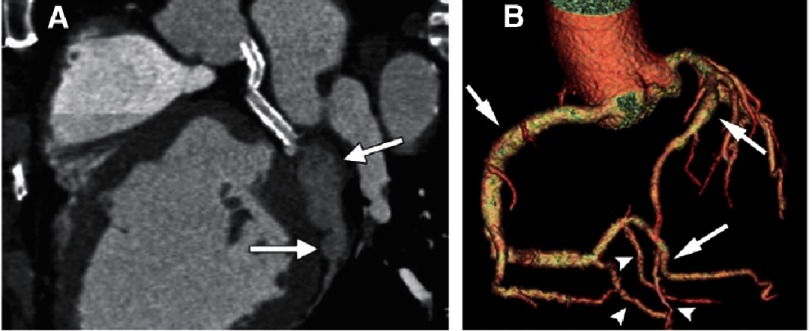
Coronary artery aneurysm compared with coronary artery ectasia. (A) Coronal reformatted image of 55 year-old man with stents in the left main and proximal circumflex coronary arteries. A saccular atherosclerotic aneurysm (arrows) is seen in the mid distal portion of the left circumflex coronary artery. (B) Volume rendered image showing ectasia in the RCA, its posterolateral branch, and the left anterior descending artery (arrows). Note normal diameters (arrowheads) of the coronaries; dilatation of the coronary arteries extends for more than 50% of the vessel length. *From Diaz-Zamudio et al.*^11^ *with permission*.

The term aneurysmal coronary artery disease (ACAD) is coined by the authors to encompass both coronary artery aneurysms (CAA) and coronary artery ectasia (CAE).^1^ CAA is defined as a localized irreversible dilatation of the coronary vascular lumen with a diameter ≥1.5 times that of the adjacent normal coronary segment.^2^ CAE describes diffuse dilatation of the coronary arteries that involves ≥50% of the length of the artery (Table 1, Figure 1). The first description of CAA is attributed to Morgagni – an Italian pathologist – in 1761. The first CAA reported in a living patient was detected by coronary angiography by Muncken et al. in 1958.^3^ CAE has been subcategorized based on its topographical extent into four types:

Type I, diffuse ectasia in two or three arteries;

Type II, diffuse ectasia in one artery and localized disease (i.e., aneurysm) in another;

Type III, diffuse ectasia in one artery only;

Type IV, localized and segmental ectatic lesions.^4^

CAA occasionally grow large enough to be called “giant” CAA defined as greater than 8 mm in diameter.^5^ The literature on ACAD is mostly limited to reports of single cases and some reviews. Although rare, ACAD can be potentially fatal if not managed judiciously and in a timely manner.^3^ The purpose of this article is to present a comprehensive overview of this group of disorders.

**Table 1:** Classification of aneurysmal coronary artery disease.

A. Focal dilatation (aneurysm)
1. Wall composition
• True aneurysm: wall composed of the 3 vascular layers
• False aneurysm: wall composed of adventitia
2. Morphology
• Saccular aneurysms: transverse > longitudinal diameter
• Fusiform aneurysms: longitudinal > transverse diameter
3. Giant aneurysm: >8 mm in diameter
B. Diffuse dilatation (ectasia)
1. Type I: diffuse ectasia in 2 or 3 vessels
2. Type II: diffuse ectasia in one vessel and aneurysm in another
3. Type III: diffuse ectasia in one vessel
4. Type IV: localized and segmental ectatic disease

## Epidemiology

The prevalence of ACAD in an angiographic series varies from 0.2 to 10%,^3^ with such wide range primarily reflecting the varied angiographic criteria used to define CAA and CEA. In the largest postmortem study, investigators showed a CAA prevalence of only 1.4%.^6^ CAE is more common than CAA.^7^ Both conditions may be seen at any age and there is no specific age predilection. The prevalence of giant CAA is very low (0.02%), with the exception of those associated with congenital coronary fistulae for which the reported prevalence is 5.9%.^8^ The right coronary artery is the most frequently affected vessel (40.4%), followed by the left anterior descending artery (32.3%), left circumflex artery (23.4%), and rarely the left main coronary artery (3.5%).^9^ Atherosclerotic or inflammatory ACAD are usually multiple and involve more than one coronary artery. In contrast, congenital, traumatic, or dissecting aneurysms are usually single.^3^ Those related to atherosclerosis usually appear later in life than those associated with congenital or inflammatory conditions.^10^

The true burden of ACAD maybe underestimated currently, but with the widespread use of coronary computed tomography and magnetic resonance coronary angiography, the rate of recognition may increase.^11^ However, data from a single tertiary care facility utilizing these technologies liberally revealed a prevalence of 2.7% - not dissimilar to the rates reported by coronary angiography and pathological studies in same institution.^12^ Important genetic and environmental influences affect the prevalence of ACAD which has been shown to be lower in Asia compared to North America and Europe. In contrast, ACAD due to Kawasaki disease is more prevalent in patients of Asian ethnicity (10.3%) than is those of Caucasian (6.9%) or African ethnicity (1.2%).^13^

## Etiology

The etiology of ACAD varies with the geographic location and the age group studied (Table 2). In western countries, atherosclerosis is the most common cause of ACAD (50%) followed by congenital (17%) and infectious causes (10%). In the Far East, Kawasaki disease is the predominant cause of ACAD.^1,2,14^ Other causes of ACAD include inflammatory arterial diseases (polyarteritis nodosa, Takayasu arteritis, Behcet’s disease, syphilis), connective tissue disorders (systemic lupus erythematosus, rheumatoid arthritis, ankylosing spondylitis, progressive systemic sclerosis), hereditary collagen defects (Marfan syndrome, Ehler-Danlos syndrome), percutaneous coronary intervention (balloon angioplasty, stenting, atherectomy), fungal infection, trauma, and primary hyperaldosteronism. Overall, atherosclerosis is the commonest cause in adults and Kawasaki disease is the commonest in children.^15,16^

**Table 2:** Common causes of aneurysmal coronary artery disease.

Etiology	Age group	Aneurysm (A) or ectasia (E)	Comments
Atherosclerosis	Adults	A/E	Most common cause of ACAD in adults. Clinical importance depends on association with significant coronary artery stenosis
Kawasaki disease	Childhood and adolescence	A	Most common cause in children and adolescents, and in the Far East
Inflammatory disorders	Young adults	E	Takayasu arteritis, systemic lupus, Behcet syndrome, polyarteritis nodosa, Reiter syndrome, psoriatic arthritis, Wegner granulomatosis, Churg-Strauss syndrome
Fistula	Any age	E	Compensatory dilatation secondary to high flow state
Coronary anomalies	Any age	E	Compensatory dilatation secondary to myocardial ischemia
Connective tissue disorders	Young adults	E	Marfan syndrome, Ehlers-Danlos syndrome, cystic medial necrosis
Mycotic	Any age	A	Infection, most commonly with staph aureus with microemboliztion to vasa vasorum or invasion of vessel wall
Trauma/iatrogenic	Adults	A	As a result of coronary interventions
Cocaine	Adult	A	Direct endothelial damage form episodic hypertension, vasoconstriction, and underlying atherosclerosis

## Pathogenesis

The pathogenesis of ACAD is incompletely understood but is likely to involve destruction of the arterial media, thinning of the arterial wall, and increased wall stress.^17,18^ With increased vascular diameter, the increased wall stress causes progressive dilatation of the affected arterial segment.^19^ Inflammation spreads from the tunica intima to the tunica media and adventitia resulting in intense transmural inflammation.^20^ Aneurysm and ectasia development is characterized by overexpression of proinflammatory cytokines and enzymes that are capable of degrading various structural proteins of the vessel wall including matrix metalloproteinases (MMPs) and enzymes that are capable of degrading elastin, collagen, proteoglycans, laminin, and fibronectin. They are present at elevated concentration in aneurysmal and ectatic segments associated with decreased levels of tissue inhibitors of matrix metalloproteinases (TIMPs). Other contributing factors include increased production of collagenases, gilatinases, and stromelysins. Genetic factors including HLA – DR B1, DR 16, DQ 2, MMP-3, 5A alleles and insertion-deletion polymorphism of the angiotensin-converting enzyme (ACE) DD genotype may increase the inflammatory response in the vascular wall with overexpression of soluble adhesion molecules including vascular cell adhesion molecule 1 (VCAM-1), intercellular adhesion molecule 1 (ICAM-1), and E-selectin. These inflammatory mediators are also increased in sera of patients with ACAD and promote the intensity of vascular inflammation.^21^ There is a significant positive correlation between the total length of ectatic segments and the level of plasma soluble VCAM-1, ICAM-1, and E-selectin.^20,22^ The MMP-3, 5A allele is associated with higher promotor activity for transcription of the gene and this allele is more common in patients with atherosclerotic ACAD than in patients with uncomplicated coronary atherosclerosis.^23^

### Atherosclerosis

Atherosclerosis is the most common cause of ACAD in adults. Atherosclerotic lesions occur predominantly at the sites of low shear stress and are more frequent in the proximal segments of the three major coronary arteries.^24^ Inflammatory cells residing in the plaque secrete cytokines that further amplify inflammation and produce proteases that destabilize the plaque and damage the extracellular matrix. Microscopic evaluation of atherosclerotic ACAD reveals typical components of atherosclerotic plaques such as mononuclear cell infiltrates, lipid deposits, cholesterol crystals, destruction of the intima and media, diffuse hyaluronization, focal fibrosis, calcification of the media, intramural hemorrhage, and sometimes a foreign body giant cell reaction.^25^ Post-stenotic transformation of kinetic energy to potential energy and imbalance between intravascular pressure and elasticity of the vascular wall may also contribute to aneurysmal dilatation and ectasia.^26^

Atherosclerotic aneurysms are usually multiple and involve more than one coronary artery as compared with congenital, traumatic, or dissecting aneurysms that are often solitary.^27^ The RCA is the most frequently involved vessel (40-61%), followed by the LAD (15–32%), and circumflex coronary artery (15–23%). Left main coronary artery involvement is rare (0.1–3.5%) and its presence is usually associated with significant two- or three-vessel coronary artery disease.^4,28,29^ Increased wall stiffness, elongation and tortuosity of the vessel leads to areas of turbulent flow which in combination with endothelial injury favors thrombus formation seen in most atherosclerotic ACAD.^30^

### Kawasaki disease

Kawasaki disease (KD), or the mucocutaneous lymph node syndrome is an acute self-limited multisystem panarteritis that may occur worldwide, but is markedly more common in Japan where it was first described by Tomisaku Kawasaki in 1967.^31^ KD is the most common cause of ACAD in children and the second most common in adults. It predominantly affects children between the age of 6 months to 7 years, although younger infants and older children may also develop the illness, often with incomplete or atypical presentation.^32^

The disease may be triggered by one or more of the common infectious agents in the genetically susceptible host. The infectious agent travels from a mucosal portal of entry through the blood stream and infects many organs and tissues including vascular wall, myocardium, respiratory tract, kidney, and pancreas. Up to 25% of untreated children will develop persistent abnormalities in the coronary arteries, but therapy with intravenous immunoglobulin (IVIG) and aspirin within the first 10 days of fever onset reduces the prevalence of coronary artery abnormalities to approximately 5%.^33^ Siblings of children with KD have a tenfold higher risk of developing the illness compared to the general population, and children whose parents had KD have a twofold increased incidence of the disease.^34^

**Figure 2.**
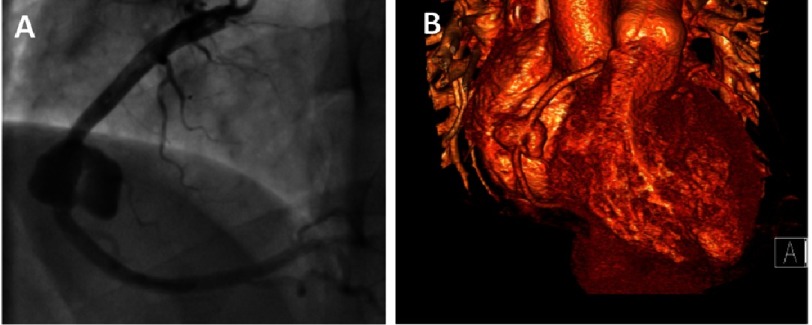
Giant coronary artery aneurysm. (A) Coronary angiogram of a 19 year-old male patient with history of Kawasaki disease during childhood showing a “giant” coronary aneurysm affecting the mid segment of the right coronary artery. (B) 3D volume-rendered reconstructed CT image of the same patient.

Inflamed tissues in acute KD show inflammatory cell infiltration of the arterial wall (monocytes, lymphocytes, and macrophages), destruction of the internal elastic lamina, necrosis of smooth muscle cells, myoinitmal proliferation, and subsequent development of aneurysms or ectasia.^35^ Among patients with KD, those with coronary artery lesions have higher plasma levels of both MMP3 and MMP9 attesting to the role of those (and other) enzymes in the destruction if collagen and elastin fibers. The immune response ultimately succeeds in controlling the putative pathogen, but damage to the coronary arteries – including aneurysmal and stenotic lesions – may have already occurred. The sizes of coronary aneurysms are classified as small (<5 mm internal diameter), medium (5-8 mm) and giant (>8 mm) (Figure 2).^32^ Regression of small and medium sized aneurysms to normal lumen diameter occurs in approximately half the cases within 2 years from the onset of illness. In contrast, obstructive lesions develop in 2 years and continue to increase over time.^36^ Stenotic lesions occur as a result of luminal myofibroblastic proliferation and thrombosis. The mechanism of aneurysmal regression involves proliferation of myofibroblasts as well as mural thrombosis. The development of coronary ischemia or stenotic lesions is rare in patients who show complete regression, at least within the first two decades after the onset of illness. However, regressed coronary aneurysms have reduced reactivity to adenosine, show myointimal thickening by intravascular ultrasound, and increased incidence of calcification on CT scans.^37,38^

The diagnosis of KD is clinical and is based on the major clinical features of the acute phase (Table 3).^39^ Coronary artery aneurysms can cause angina, myocardial infarction (MI), and sudden death. The risk of MI is highest in the first year of the disease, but may occur many years later particularly if coronary artery stenosis worsens. Recurrence of MI occurs in about 20% of children.^36^ Aneurysms of other arteries including the axillary, common iliac, internal iliac, renal, mesenteric, and internal thoracic arteries were observed in 1% of patients.^40^ Renovascular hypertension may develop in patients with renal artery lesions.^41^ Other cardiovascular manifestations of KD include mild mitral and aortic regurgitation, aortic root dilatation, pericarditis, and myocarditis. Systolic function virtually always recovers to normal in absence of ischemic heart disease.^42,43^

**Table 3:** Clinical features of the acute phase of Kawasaki disease.

Characteristic combination of:
- Prolonged high fever
- Rash
- Stomatitis/conjunctivitis
- Erythema of the hands and feet with late peeling
- Lymphadenopathy

Patients can first present in adulthood with angina pectoris or myocardial infarction several decades after the onset of the disease. The coronary sequelae of KD may be an important cause of ischemic heart disease in young adults, particularly those under 40 years of age. In one study, KD was the cause of myocardial infarction in 5% of adults under the age of 40.^44^ Therefore, a history of KD should be sought in adults with ACAD on the absence of atherosclerotic risk factors and/or generalized atherosclerotic disease. Reports of adult cases of KD are viewed with skepticism. In adults, the incidence of specific diagnostic criteria is similar to that in children,^45,46^ however arthralgia, gastrointestinal complications and liver function abnormalities are more common than in children.^47^ The incidence of ACAD in adults is reported to be lower than in children (5 in 57 reported adult cases) partly because of the difficulty in visualizing adult coronary arteries with transthoracic echocardiography.^48^ It is uncertain whether ACAD that are persistent or have regressed are more predisposed to atherosclerosis in adulthood.

Coronary artery lesions in the acute stage of KD are detected by 2D echocardiography using standard views. However, echocardiography is less reliable for detection of distal aneurysms or stenotic lesions. When echocardiographic images are inadequate, for example in adolescents and adults, ultrafast computed tomography (CT) and magnetic resonance imaging (MRI) are useful noninvasive techniques for evaluation of coronary artery architecture.^49,50^ Selective coronary angiography (CAG) is the “gold standard” for evaluation of the presence and severity of coronary artery abnormalities in KD patients. Indications for CAG include the presence of significant coronary aneurysms by 6-12 months after the onset of illness when CT or MRI cannot provide high quality information at lower risk; evidence of ischemic heart disease requiring revascularization; and when there is a need to delineate precise coronary anatomy for therapeutic decisions, for example the type of antithrombotic therapy.^36^ Follow up CAG is often needed for evaluation of stenotic or obstructive lesions of the coronary arteries. Intravascular ultrasound (IVUS) can provide information about myointimal thickening, generally present in arterial segments that were larger than 4 mm early in the disease.^51^ Measurement of fractional flow reserve (FFR) may provide guidance for decisions about revascularization. It is particularly important to accurately assess the presence of myocardial ischemia during follow up. When feasible, exercise stress testing is preferable to pharmacological stress testing because it mimics the physiology of daily activities. Stress echocardiography is preferable to nuclear or PET stress testing to diminish exposure to ionizing radiation. In children who are too young to exercise, dobutamine or adenosine stress MRI or other pharmacological stress testing modalities are useful.^36^

Current management of acute KD is based on prospective, controlled, multicenter treatment trials that clearly demonstrated the efficacy of intravenous immunoglobulins and high-dose aspirin to halt inflammation and reduce the likelihood of the development of coronary abnormalities when administered by the tenth (and preferably the seventh) day of illness.^52^ IVIG resistant disease may need corticosteroids, infliximab, cyclosporine, cyclophosphamide, methotrexate and plasmapharesis.^3^ Long-term management depends on the degree of coronary arterial involvement. All children with ACAD are treated with antiplatelet-dose aspirin. In patients with giant aneurysms, who are at the highest risk of coronary thrombosis, anticoagulation with warfarin or low-molecular weight heparin is added to aspirin.^53,54^ Among children with aneurysms just below the threshold for giant or those with giant aneurysms in whom anticoagulation cannot be safely managed, a thinopyridine is often added to aspirin. Children who required thrombolysis for coronary thrombosis are treated with triple therapy (anticoagulant, aspirin, and a thienopyridine) for a short period balancing the risk of recurrent thrombosis with that of bleeding.^3^ Strategies for acute reperfusion therapy are based upon those used in adults. However, mechanical restoration of flow is not feasible in the youngest children, for whom thrombolytic therapy with tPA is the standard therapy.^39^ Coronary revascularization with percutaneous coronary intervention (PCI) or coronary artery bypass grafting (CABG) are indicated in the presence of ischemic symptoms, inducible myocardial ischemia on stress testing, or more than 75% stenosis of proximal coronary arteries. Children with severe left ventricular dysfunction or complex coronary lesions with multiple ostial or long segment stenoses are to be considered for CABG. Arterial grafts appear to grow with the child and are therefore preferable to venous grafts. Graft longevity is superior in children older than 12 years of age. In a leading Centre for such procedures in Japan, 95% of patients were alive and 60% had patent grafts without reintervention 25 years after CABG.^55^ End-stage ischemic cardiomyopathy that cannot be improved by revascularization procedures may need cardiac transplantation.^56,57^

### Polyarteritis nodosa

Polyarteritis nodosa (PAN) was first described in 1866 by Kussmaul and Maier. It is a multisystem nectrotizing vasculitis of small- and medium-sized muscular arteries in which involvement of renal and visceral arteries is characteristic. PAN does not affect pulmonary arteries, although the bronchial vessels may be affected. PAN affects 2-6 people per 100,000 per year. The lesions are segmental and tend to involve bifurcation and branching points of arteries. They may spread circumferentially to involve adjacent veins. Coronary artery involvement occurs in 76% of patients and ranks second after renal arteries (85%).^58^ Arteritis, aneurysms, and thrombosis of the coronary arteries are known complications of the disease. In the acute stages of the disease, polymorphnuclear neutrophils infiltrate all layers of the vessel wall and perivascular areas, which results in intimal proliferation and degeneration of the vessel wall. Mononuclear cells infiltrate the area as the lesions progress to the subacute and chronic stages. Fibrinoid necrosis of the vessel ensues with compromise of the lumen, thrombosis, infarction of the tissues supplied by the involved vessel, and in some cases hemorrhage. As the lesions heal, further collagen deposition may lead to more compromise of the vessel lumen. Aneurysmal dilations – up to one cm in size – along the involved arteries are characteristic of PAN.

The presence of PAN-like vasculitis in patients with hepatitis B together with the isolation of circulating immune complexes composed of hepatitis B antigen and immunoglobulin strongly suggests an immunological role in the pathogenesis of this disease.^59^ A PAN-like vasculitis has also been reported in patients with hepatitis C.^60^

Patients usually present with fever and vague symptoms such as weakness, malaise headache, abdominal pain, and myalgia that can rapidly progress to a fulminant illness. Specific complications related to the vascular involvement within a particular organ system may also dominate the presenting clinical picture as well as the entire course of illness. There are no diagnostic serological tests for PAN. Antibodies against myeloperoxidase or proteinase-3 (ANCA) are rarely found.^61^ The diagnosis is based on the demonstration of characteristic findings of vasculitis on biopsy specimens of involved organs. In the absence of easily accessible tissue for biopsy, the arteriographic demonstration of involved vessels particularly in the form of aneurysms of small- and medium-sized arteries in the renal, coronary, hepatic, and visceral vasculature is sufficient to establish the diagnosis. This should consist of catheter-directed angiography because computed tomography and magnetic resonance angiography do not have sufficient resolution at the current time to visualize the vessels affected.^60^ Aneurysms need not always be present, and arteriographic findings may be limited to stenotic segments and obliteration of vessels. Biopsy of symptomatic organs such as nodular skin lesions, nerve, muscle, and painful testes may provide the highest diagnostic yield.

The prognosis of untreated PAN is extremely poor, with a reported 5-year survival rate of 10-20%. Death is usually the result of cardiovascular and gastrointestinal complications. Treatment with a combination of prednisone and cyclophosphamide increases survival substantially. In patients with hepatitis B, antiviral therapy represents an important part of therapy and has been used in combination with glucocorticoids and plasma exchange. Aneurysms may resolve over time as remission occurs. However, following successful treatment, relapse of the disease occurs in 10-20% of patients.^60^

### Takayasu arteritis

Takayasu arteritis (TA) is an inflammatory disease of medium- and large-sized arteries characterized by a strong predilection for the aortic arch and its branches. It has an estimated annual incidence rate of 1.2 to 2.6 cases per million. It primarily affects females with a female to male ratio of 8:1 in adults. However, the ratio is significantly lower in childhood (2:1).^62^ Although it is more common in Asia, it is neither racially nor geographically restricted.

The most commonly involved arteries in descending order of affection are the subclavian (93%), common carotid (58%), abdominal aorta (47%), and renal (38%) arteries. The coronary arteries are involved in less than 10% of cases.^60^ The pulmonary artery may also be involved. The incidence of aneurysms in patients with TA varies between 18% and 24%, however coronary aneurysms are extremely rare.^63^

TA is a panarteritis with inflammatory mononuclear cell infiltrates and occasionally giant cells. There is marked intimal proliferation and fibrosis, scarring and vascularization of the media, as well as disruption and degeneration of the elastic lamina. Narrowing of the lumen occurs with or without thrombosis. ACAD develops as a result of vascular wall weakness and arterial hypertension that frequently coexists due to renal artery involvement. However, most coronary lesions are stenotic,^64^ involve the coronary ostia, and may be focal or diffuse.^65^

The diagnosis of TA should be strongly suspected in a young woman who develops a decrease or absence of peripheral pulses, discrepancy in blood pressure, and carotid bruit. Fever, malaise, arthralgias, and weight loss may occur months before vascular involvement becomes apparent. These symptoms may merge into those related to vascular compromise and organ ischemia. Hypertension occurs in 32% of 93% of patients and contributes to cardiac, renal, and cerebral injury.

The diagnosis is confirmed by the characteristic pattern of arteriography which includes irregular vessel walls, poststenotic dilatation, aneurysm formation, occlusion, and evidence of increased collateral circulation. Complete aortic arteriography by catheter-directed angiography, computed tomography, or magnetic resonance arteriography should be obtained to fully delineate the distribution and degree of arterial disease. Tissue is rarely readily available for examination. The course of the disease is variable, and although spontaneous remission may occur, TA is most often chronic and relapsing. Disease-related mortality most often occurs from congestive heart failure, cerebrovascular events, myocardial infarction, aneurysm rupture, or renal failure. The combination of glucocorticoid therapy for acute signs and symptoms and an aggressive surgical and/or angioplastic approach to stenotic and dilated vessels has markedly improved outcome and decreased morbidity. Drug-eluting stents have also been used to effectively treat stenotic lesions.^66,67^ Surgical correction should be undertaken only when the vascular inflammatory process is well-controlled with medical therapy.^68^ In individuals who are refractory to or unable to taper glucocorticoids, methotrexate and anti-tumor necrosis factor (anti-TNF) therapy has yielded encouraging results.^60^

### Coronary artery fistulas

Most reported cases of congenital ACAD are associated with coronary artery fistulas and are often identified in young populations. A coronary fistula constitutes a connection between a coronary artery and a cardiac chamber or great vessel bypassing the myocardial capillary bed. Although most coronary fistulas are congenital in origin, they can also develop as a complication of valve or coronary surgery, or cardiac biopsy.^69,70^

Coronary artery fistulas may arise from many sites; the more proximal the site where the feeding artery arises from the main coronary artery, the more dilated the feeding artery tends to be.^71^ Fistulas originate from the right coronary artery in 52% of cases; the left anterior descending artery in 30% of cases; and the left circumflex artery in 18% of cases.^72^ In 90% of patients, the drainage is to the right cardiac chambers resulting in volume overload of the pulmonary vascular bed, left atrium and left ventricle. Drainage to the left cardiac chambers spares the pulmonary vasculature.

ACAD may also be the consequence of coronary anomalies, particularly the origin of right coronary artery from the pulmonary artery (ARCAPA) or the anomalous origin of the left coronary artery from the pulmonary artery (ALCAPA or Bland-White-Garland syndrome). In the most common form of ALCAPA, the left main coronary artery arises from the main pulmonary artery and the normal origin right coronary artery becomes ectatic as a compensatory mechanism. Important collateral circulation between the right coronary artery and the left coronary circulation and a coronary steal phenomenon into the pulmonary artery – with myocardial ischemia in the territory of the abnormal vessel – may develop.^73,74^

### Miscellaneous

In addition to the disorders mentioned above, some other diseases are involved in the production of ACAD. Systemic lupus erythematosus (SLE) commonly causes vasculitis mediated by autoantibodies and immune complexes.^60^ In most patients, autoantibodies are present years before the clinical presentation. 90% of patients are women of child-bearing age, but people of all genders, ages and ethnic groups are susceptible. Generally, coronary aneurysms are large and proximal, and occur in the setting of other obvious clinical markers of the disease. This includes cutaneous rashes, arthritis, pancytopenia, serositis, organic brain syndromes, and nephritis. Patients are at an increased risk of myocardial infarction, usually due to accelerated atherosclerosis.^60^ Diagnosis depends on published criteria with ≥ 4 criteria carrying 93% specificity and 92% sensitivity for SLE.^60^

Marfan syndrome (MFS) can also cause ACAD. More than 90% of patients with MFS have a mutation in the gene coding for fribrillin-1 (FBN 1). A few have a mutation in the gene coding for TGF-β receptor 2 (TGFBR2). The severity of the phenotype cannot be predicted from the nature of the mutation. Some of the manifestations of MFS have been shown to arise from alterations in binding sites that modulate TGF-β bioavailability during development of the skeleton and other tissues. The resulting increased TGF-β signaling contributes to aneurysm formation. Although TGF-β can be inhibited by angiotensin II-type I-receptor antagonists such as losartan and can prevent aortic aneurysm development in a mouse model of MFS, this could not be replicated in human trials.^75^

ACAD related to coronary interventions is rare but has been reported in an increasing number of reports of balloon angioplasty, stenting, atherectomy, and laser angioplasty. ACAD may occur from 3 days up to 9 days after stent implantation. Within the first 9 months, the reported incidence of coronary aneurysms after bare metal stents (0.3 to 3.9%) is similar to that after drug eluting stents (0.2 to 2.3%), but it remains unclear whether late aneurysm formation occurs more frequently with either type of stents. ACAD complicating coronary intervention may be related to mechanical damage to the arterial wall, inflammatory and allergic reaction to the deployed stents (stent platform, carrier polymer, and/or antiproliferative drug), and delayed healing with incomplete endothelialization.^16,76^

Rarely, mycotic aneurysms caused predominantly by staphylococcus aureus complicate stent deployment and the local immunosuppressive effects of eluted drugs may increase the incidence of such infectious aneurysms. Clinical presentation varies from asymptomatic to ischemic manifestations and occasionally aneurysmal rupture and hemorrhagic pericarditis. Mycotic aneurysms typically present with fever and bacteremia.^77–79^ Three types of aneurysms complicating coronary interventions are recognized: Type I, demonstrates pseudoaneurysms with rapid early growth related to arterial injury during the interventional procedure; type 2, refers to aneurysms detected incidentally during angiography ≥ 6 months after the procedure; and type III, are mycotic or infectious aneurysms.

**Figure 3.**
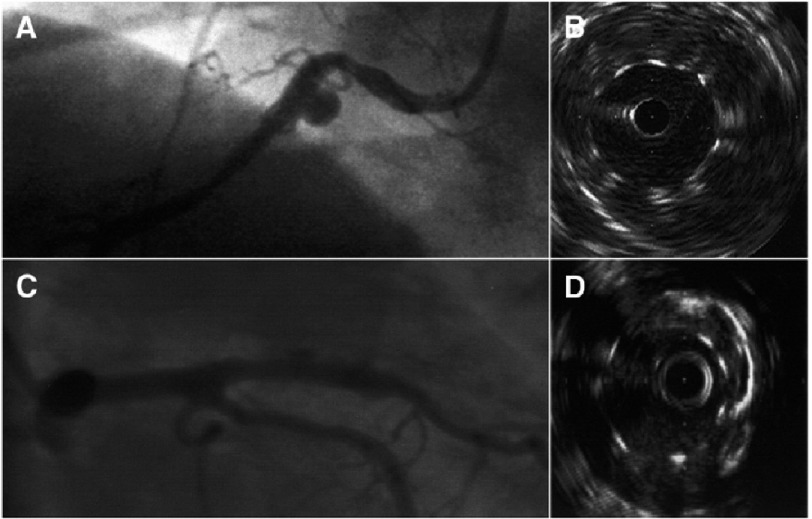
Angiographic and IVUS images of coronary artery aneurysm. (A and B) Coronary artery aneurysm detected by coronary angiography and intravascular ultrasound (IVUS) 8 months after bare-metal stent implantation. (C and D) Coronary artery aneurysm detected with coronary angiography and IVUS 3 months after drug-eluting stent implantation. *From Aoki et al.*^16^ *with permission*.

Coronary angiography and IVUS are the gold standard for diagnosis (Figure 3). Computed tomography, coronary magnetic resonance imaging and real time 3-dimensional echocardiography can also be used to detect and follow up certain coronary aneurysms noninvasively.^80–82^ The treatment of ACAD complicating coronary interventions remains speculative in the absence of clinical trials. It should be individualized depending on the aneurysm size, growth, pathophysiology and symptoms. For large pseudoaneurysms (type I) and true aneurysms (type II – at least twice the reference vessel diameter), especially if symptomatic, interventional or surgical treatment is proposed. The threshold for treatment may be lower for pseudoaneurysms than for true aneurysms because of the presumed greater likelihood for rupture. Immediate surgical treatment should be implemented for any confirmed infected aneurysm (type III). Concerns related to stent graft treatment include closure of contiguous side branches arising to the aneurysm site, stent thrombosis, and restenosis. Placing coronary coils behind stents to thrombose the aneurysm sac can also be challenging. Finally, long-term antithrombotic drug therapy including aspirin and clopidogrel is necessary to reduce the risk of stent thrombosis. Since some aneurysms – even large ones – might resolve spontaneously, further investigation is necessary to determine the natural history of and best therapy of such aneurysms.^16^

Mycotic coronary aneurysms may develop unrelated to coronary intervention secondary to microembolization to the vasa vasorum, direct pathogen invasion of the arterial wall, or immune complex deposition.^83^

Cocaine abuse is associated with increased prevalence of ACAD (30.4%). Such patients are at increased risk of myocardial infarction, arrhythmias, hypertension and cerebrovascular disease. Several mechanisms have been proposed for the development of ACAD related to cocaine abuse including endothelial damage, vasoconstriction, and underlying atherosclerosis.^84^

## Clinical manifestations and complications

Most patients with ACAD remain asymptomatic, and coronary abnormalities are incidentally discovered during coronary imaging or at necropsy.^28^ When symptoms occur, the manifestations depend on the underlying cause. In cases associated with atherosclerosis, clinical manifestations are similar to those seen in coronary artery disease including angina pectoris, dyspnea, myocardial infarction, and sudden death.^85^ Occasionally, a systolic murmur is heard over the precordium. Other factors that can contribute to symptoms are the extent of atherosclerosis, the presence of poor distal vessel runoff, and the development of complications such as distal embolization with myocardial infarction, rupture with consequent hemopericardium and cardiac tamponade, as well as coronary thrombosis, dissection and vessel compression.^11^ An association with abdominal aortic aneurysms and hypertension has been reported.^2^

## Diagnosis

ACAD can be diagnosed by noninvasive techniques including transthoracic echocardiography, transesophageal echocardiography, computed tomography (CT), and magnetic resonance angiography (MRA). Tools that do not involve exposure to radiation are recommended for follow up, particularly in children with Kawasaki disease.^86^ Three-dimensional evaluation with these techniques helps to provide an easy understanding of complex anatomical structures, and allows analysis of the lumen and vessel wall as well as identification of thrombi and associated plaques. However, these noninvasive modalities mostly allow the investigation of only the proximal segments of the coronary arteries.

Coronary arteriography remains the “gold standard” for the evaluation of ACAD. It provides adequate information regarding the size, shape, location, and the number of existing anomalies.^87^ Angiographic signs of turbulent flow include delayed antegrade dye filling, a segmental back flow phenomenon, and local stagnation of dye in dilated coronary segments.^88^ IVUS and Doppler flow wire estimation of fractional flow reserve can be incorporated into invasive angiography. Nonetheless, the true size of the aneurysm may be underestimated by angiography or the ACAD may be totally missed when it is occluded or contains substantial thrombi or plaques.^89^

## Treatment

Although several investors have reported data on ACAD, there are no controlled clinical trials to evaluate the optimal therapy for those disorder because of their rarity. Medical therapy generally consists of attempts to prevent thromboembolic complications in patients with aneurysmal arteries who are at increased thrombotic risk using antiplatelet and anticoagulant medications.^90^ Treatment strategies to improve outcomes in vasculitis-induced ACAD involve the use of immunosuppressive therapy to combat the underlying inflammatory process. As previously mentioned, the use of high-dose intravenous therapy with immunoglobulins together with aspirin reduces the rate of occurrence of coronary lesions in patients with Kawasaki disease.^91,92^ Because elevated MMP-3 levels likely contributes to the development of ACAD, statins and corticosteroids could be beneficial in inhibiting such cytokines.^93^ Trimetazidine can also improve coronary flow by increasing adenosine levels. However, administration of nitroglycerin and nitrate derivatives may induce further coronary dilatation and should be avoided.^94^

**Figure 4.**
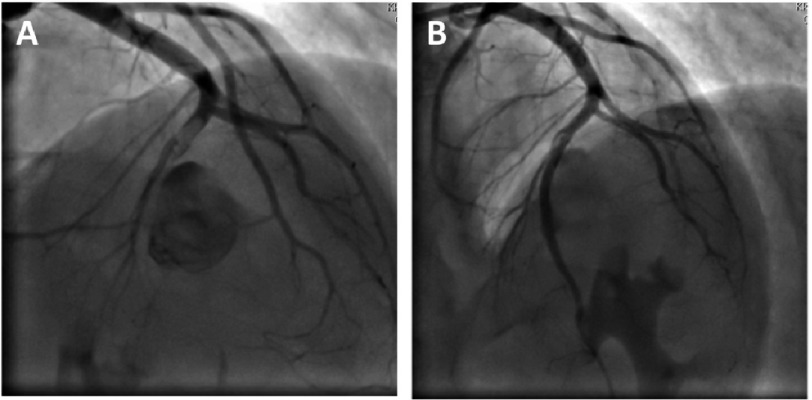
Percutaneous treatment of a mid-LAD aneurysm. (A) Coronary angiogram of a 22 year-old male patient presenting with acute anterior ST-elevation myocardial infarction. A giant aneurysm (etiology unknown) is seen in the mid segment of the left anterior descending (LAD) coronary artery which is subtotally occluded after the aneurysmal segment. (B) TIMI 3 flow restored in the distal LAD after primary percutaneous coronary intervention using a 3.0 × 28 mm PTFE-covered stent with cessation of flow into the aneurysm.

Percutaneous treatment with polytetrafluoroethylene (PTFE)-covered stents has gained popularity due to their ability to effectively limit expansion of coronary aneurysms by reducing blood flow within the dilated segments thereby preventing their rupture (Figure 4). Some investigators suggested that the use of PTFE-covered stents should be limited to patients whose aneurysms are <10 mm in diameter and those with a fistula that needs closure.^95,96^ Other percutaneous options include coil embolization and autologous saphenous vein-covered stent grafting.

Surgical management is appropriate for ACAD three times or larger than the original/reference diameter, patients with obstructive coronary artery disease, and those with evidence of embolization leading to myocardial ischemia despite antithrombotic therapy. Surgical techniques including aneurysm ligation, resection, marsupialization with interposition graft, and coronary artery bypass surgery with ligation or resection of the aneurysm.^97^

## Prognosis

The prognosis of ACAD remains controversial.^98,99^ The prognosis may be directly related to the severity of concomitant obstructive coronary disease. In the coronary artery surgery study (CASS) registry, there was no difference in the 5-year survival rate between patients with or without aneurysms who had obstructive coronary artery disease.^100^ However, in another large cohort, ACAD was an independent predictor of mortality with an overall 5-year survival rate of 71%. Nevertheless, no significant association was found between aneurysm size and mortality.^98^ Rupture of aneurysms is rare and unpredictable.^101^ The prognosis for aneurysms complicating coronary intervention is generally favorable. In KD, 50% of acute cases showed regression of coronary aneurysms on follow up 1-2 years after the onset of disease, and nearly all of them were event-free on long-term follow up. Factors associated with spontaneous regression include age <1 year, saccular morphology, and distal location. Regression is unlikely in giant aneurysms or those of several years’ duration.^40^

## Conclusion

ACAD is an uncommon finding, however with the advent of modern noninvasive imaging techniques, such lesions are more frequently encountered. Atherosclerosis is the most common cause of ACAD in adults, while Kawasaki disease is the most common cause in the Far East and in children or adolescents. Most patients are asymptomatic and ACAD is discovered incidentally. The diagnostic approach depends on the clinical scenario. Management varies from medical treatment to stent insertion and surgical repair or resection. Experimental models – similar to those used in studying abdominal aortic aneurysms – can improve our understanding of the pathogenesis and natural history of ACAD.
